# When *WAS* Gene Diagnosis Is Needed: Seeking Clues Through Comparison Between Patients With Wiskott-Aldrich Syndrome and Idiopathic Thrombocytopenic Purpura

**DOI:** 10.3389/fimmu.2019.01549

**Published:** 2019-07-09

**Authors:** Ying-Ying Jin, Jing Wu, Tong-Xin Chen, Ji Chen

**Affiliations:** ^1^Department of Rheumatology/Immunology, Children's National Medical Center, Shanghai Children's Medical Center, Shanghai Jiao Tong University School of Medicine, Shanghai, China; ^2^Division of Immunology, Institute of Pediatric Translational Medicine, Children's National Medical Center, Shanghai Jiao Tong University School of Medicine, Shanghai, China; ^3^Department of Dermatology, Children's National Medical Center, Shanghai Children's Medical Center, Shanghai Jiao Tong University School of Medicine, Shanghai, China

**Keywords:** Wiskott-Aldrich syndrome, idiopathic thrombocytopenic purpura, primary immunodeficiency, gene mutation, hematopoietic stem cell transplantation

## Abstract

**Background:** Wiskott-Aldrich syndrome (WAS) is a rare and severe X-linked disorder with variable clinical phenotypes correlating with the type of mutations in the *WAS* gene. The syndrome is difficult to differentiate from idiopathic thrombocytopenic purpura (ITP) before genetic diagnosis. We retrospectively reviewed patients suspected to have WAS who were referred to our hospital from 2004 to 2016 and compared the clinical features and laboratory examination of genetically confirmed WAS patients and of patients diagnosed with ITP in order to seek some clues to distinguish WAS and ITP before genetic diagnosis.

**Methods:** Seventy-eight children suspected to have WAS from 78 unrelated families were enrolled in this study. The clinical data and laboratory examination of children were reviewed in the present study. The distribution of lymphocyte subsets from peripheral blood was examined by how cytometry. *WASP* mutations were identified by direct sequencing of PCR-amplified genomic DNA.

**Results:** Forty-two patients were finally diagnosed with WAS genetically. The median onset age of these patients was 1 month (range: 1 day−10 months). The median diagnosis lag was 4.6 months (range: 0 months−9.42 years). Fifteen patients (35.71%) had positive family histories. More than half of the patients (*n* = 23, 54.76%) had diarrhea. Twenty-three (54.76%) had pneumonia, 7 with severe symptoms. Major bleeding events included skin spots or petechiae (*n* = 27, 64.29%), per-rectal bleeding (*n* = 21, 50.00%), epistaxis (*n* = 7, 16.67%) and intracranial bleeding (*n* = 2, 4.76%). Twenty-nine patients (69.05%) had eczema, and one patient had a drug allergy. Three patients had autoimmune diseases, among whom 2 had autoimmune hemolytic anemia and one had autoimmune hemolytic anemia and IgA nephropathy. A total of 42 mutations in *WASP* were identified, including 19 novel mutations. Eight patients received hematopoietic stem cell transplantation (HSCT) and all survived. Compared with the 30 patients diagnosed with ITP, the WAS patients had higher EOS counts and elevated IgE level, increased NK cell numbers but fewer CD8^+^T lymphocytes.

**Conclusion:** The *WAS* gene diagnosis should be considered in all males with ITP-like features, especially for patients with a very early onset age, decreased MPV (<6.5 fl), higher EOS counts and elevated IgE level, increased NK cell number, diminished CD8^+^T lymphocyte count.

## Introduction

Wiskott-Aldrich syndrome (WAS) is a rare X-linked primary immunodeficiency disease (PID) characterized by thrombocytopenia, eczema, recurrent infections, and an increased incidence of autoimmunity and malignancies (1, 2).

The syndrome was first described by Alfred Wiskott in 1937 (3) and Robert Aldrich in 1954 (4), and it was thus named *WAS* after the two physicians who described it. The disease is caused by loss-of-function mutations in the *WAS* gene, which was identified in 1994 and is located on the X chromosome (position Xp11.22-p11.23) (5). The *WAS* gene encodes a 502-amino-acid intracellular protein expressed exclusively in the cytoplasm of hematopoietic cells (6). *WAS* gene mutation impairs expression and/or function of the WAS protein (WASp), which plays a key role in the functions of T cells, natural killer (NK) lymphocytes, and dendritic cells (7). WASp is a multifaceted protein associated with several clinical disorders. Absent WASp causes classic WAS described above. Mutations that result in decreased, but not absent, protein expression cause the milder disease X-linked thrombocytopenia (XLT) that is characterized mainly by thrombocytopenia and sometimes is associated with mild eczema and immunodeficiency (8). Furthermore, a third disorder termed X-linked neutropenia (XLN), characterized by neutropenia and variable myelodysplasia, has been attributed to activating mutations in the GTPase-binding domain (GBD) of WASp (9).

The long-term prognosis of classic WAS is generally poor, with hematopoietic stem cell transplantation (HSCT) remaining the only curative choice (10). Frequent complications of WAS entail fatal intracranial bleeding, severe and recurrent infections, autoimmune diseases, and malignancies throughout adulthood (1). Early and timely diagnosis and treatment are critical for patients with WAS to improve outcomes.

However, WAS is sometimes difficult to differentiate from other thrombocytopenic disorders and is often initially diagnosed as idiopathic thrombocytopenic purpura (ITP), which can lead to inappropriate treatment and delays to life-saving definitive therapy (11–13). WAS is traditionally differentiated from ITP by the small size of WAS platelets (14). In practice, microthrombocytopenia may occasionally not be present and some WAS patients have normal mean platelet volume (MPV) (15, 16) or macrothrombocytopenia (2, 17). Therefore, differentiation of the disease from ITP is not always easy on clinical grounds. We retrospectively reviewed patients suspected of WAS referred to our hospital from 2004 to 2016 and compared the clinical features and laboratory examination between genetically confirmed *WAS*/XLT patients and patients diagnosed with ITP to seek some clues to distinguish WAS/XLT and ITP clinically before genetic diagnosis. Such clues may make the diagnosis of WAS/XLT prompt and timely and provide WAS/XLT patients earlier and better treatment in order to improve their survival rate.

## Materials and Methods

### Patients

Children's National Medical Center, Shanghai Children's Medical Center, is a teaching hospital of Shanghai Jiao Tong University School of Medicine and Department of Rheumatology and Immunology is one of the biggest referral centers for PID in China.

During the period from January 2004 to December 2016, 725 patients diagnosed with PID in our center and 78 children suspected to have WAS from 78 unrelated families were enrolled in this study. All of the patients were either first diagnosed and treated in our center or referred from other departments in our hospital or other hospitals. Patients were initially diagnosed as WAS based on typical clinical and laboratory findings, including thrombocytopenia, and/or small platelets, petechiae, bruises, recurrent, or severe infections and eczema (18). Patients were assigned a score based on their clinical severity, using a previously described scoring system (19, 20). Informed consent was obtained from each patient's parent or guardian before enrollment in the study.

### Data Collection

Clinical information was retrospectively collected from the patients' medical records, including clinical manifestations (recurrent infections, autoimmune diseases, or malignancy diseases), onset age, diagnosis age, parental consanguinity, family history of immunodeficiency, vaccination and allergic history, complications, laboratory tests, and treatments.

### Patient Samples and Cell Preparation

Heparinized or EDTA-treated peripheral blood was obtained by venipuncture from patients suspected with WAS and family members of the patients. Informed consent was obtained in each instance prior to analysis from children's parents or other legal guardians. The study protocol was approved by the Ethics Committee of Children's National Medical Center, Shanghai Children's Medical Center, Shanghai Jiao Tong University School of Medicine.

### Evaluation of Immunological Function

Routine evaluation of immunological function included the detection of immunoglobulins G, A, M, and/or E and analysis of lymphocyte subsets. The level of Immunoglobulins G, A, M, and E in serum were detected by nephelometry. While lymphocyte subsets were analyzed by a FACSCalibur flow cytometer (Becton Dickinson, USA).

### Flow Cytometry Analysis

Intracellular detection with anti-WASp monoclonal antibody was performed as described before (21). Peripheral blood mononuclear cells (PBMCs) were isolated by Ficoll-Hypaque (Axis-Shield, Norway) density gradient centrifugation and washed twice with phosphate-buffered saline (PBS). Cytofix/Cytoperm solution from CytoStain Kits (Pharmingen, San Diego, CA) was added to suspended 2 × 10^6^ PBMCs in each tube at 4°C for 20 min. After washing twice in Perm/Wash solution, they were incubated with 0.25 mg/ml purified mouse anti-human WASp mAb (BD Pharmingen) or isotype-matched control mouse IgG2a mAb (BD Pharmingen) at 4°C for 30 min and washed twice. Subsequently, phycoerythrin (PE)-conjugated goat anti-mouse IgG2a (BD Pharmingen) was added to all cells and reacted at 4°C for 30 min. WASp expression in PBMCs was analyzed by FACS Calibur, using CellQuest software (Becton Dickinson).

### Mutation Analysis of the *WAS* Gene

*WAS* gene mutation was investigated in patients, and the carrier status of their mother, female siblings, or maternally related female family members was determined also.

Genomic DNA was isolated from PBMCs using the RelaxGene Blood DNA System (Tiangen Biotech, Beijing, China) according to the manufacturer's instructions. And the DNA was amplified by PCR using synthetic oligonucleotide primers designed to amplify the *WAS* gene. Amplified DNA products were directly sequenced as described previously (22). Homology analysis with the *WAS* reference sequence was performed by the NCBI program BLAST (http://www.ncbi.nlm.nih.gov/BLAST/). When a novel missense mutation was identified, genotyping of 100 alleles was performed to rule out the possibility of being a polymorphism.

### Statistical Analysis

Statistical analysis was performed using GraphPad Prism 7 software (GraphPad Software, Inc., San Diego, CA). For measurement data, data were presented as the mean ± SD or median. Comparisons between two groups were carried out with the use of Student's *t*-test for data of normal distribution and non-parametric rank sum test (Mann-Whitney *U*-test) for abnormal distribution data. Statistical analysis on enumeration data was expressed as percentage through chi-square analysis. *P* < 0.05 was considered significant.

## Results

During the period from January 2004 to December 2016, 725 patients with PID were referred to our hospital. Forty-two (5.79%) were finally diagnosed as WAS/XLT with genetic confirmation.

### Demographics of WAS/XLT Patients

All patients were ethnic Chinese, and all were males. The origins of our patients covered 11 of the 34 provinces in China. Zhejiang, Jiangsu, Anhui and Jiangxi provinces, and Shanghai covered 57.14% (*n* = 24) of the total patient sample. This distribution seemed to be consistent with the convenience of coming to our hospital.

The median onset age of these patients was 1 month (range: 1 day−10 months). Age of onset was not correlated with the disease severity score (*p* = 0.99). The median diagnosis lag was 4.6 months (range: 0 months−9.42 years). Fifteen patients (35.71%) had positive family histories of previous early death of family members, but the diagnosis lag had no difference between patients with positive and negative family histories (*p* = 0.13).

### Clinical Features of WAS/XLT Patients

#### Infections

More than half of the patients (*n* = 23, 54.76%) had diarrhea, 2 due to rotavirus (P25 and P31) and 1 to bacilli (P32). Pneumonia was the most common infection in our patients. Twenty-three (54.76%) had pneumonia, 7 with severe symptoms (P7, P9, P17, P21, P27, P33, and P42), and 2 even deteriorated into respiratory failure (P33 and P42) and 2 into septicemia (P17 and P27). One patient died (P7). Other infectious diseases included otitis media (*n* = 3, 7.14%), thrush (*n* = 3, 7.14%), lymphadenitis, CMV and EBV infections (Table 1).

**Table 1:** Clinical features of 42 WAS patients.

**Patients**	**Family history**	**Infections**				**Bleeding**				**Autoimmune diseases**	**Eczema**	**Therapy**		**Status**
		**Diarrhea**	**URI**	**Pneumonia**	**others**	**Skin**	**GI**	**Nasal cavity**	**Head**			**IVIG**	**HSCT**	
1			+								+			Alive
2		+		+		+					+			Alive
3	+		+	+	Lymphadenitis, otitis media, mumps									Alive
4	+	+		+			+				+			Loss to follow-up
5	+	+		+	EBV infection		+	+	+		+	+	+	Alive
6			+							AIHA	+	+	+	Alive
7	+	+		+, Severe			+							Died at 2m due to pneumonia
8		+		+			+	+		AIHA IgA nephropathy		+		Alive
9				+, Encapsulated pyogenic pneumothorax	Purulent meningitis	+	+				+			Alive
10		+	+			+					+			Alive
11						+					+		+	Alive
12	+	+				+					+	+		Alive
13						+	+					+	+	Alive
14		+				+					+	+		Loss to follow-up
15		+		+	Thrush		+				+	+		Alive
16		+	+			+								Alive
17				+, Severe	Septicemias	+					+	+		Died at 7m due to severe infection
18	+	+		+			+		+		+			Died at 2m due to intracranial hemorrhage
19	+		+		Otitis media	+	+				+			Alive
20	+			+		+					+	+		Alive
21				+, Severe		+		+			+	+	+	Alive
22	+	+				+	+				+	+	+	Alive
23		+	+			+	+					+		Alive
24		+		+		+	+	+			+	+		Alive
25	+	+, RV		+			+	+			+	+		Loss to follow-up
26					Thrush		+					+		Loss to follow-up
27				+, Pulmonary abscess	Septicemia	+					+	+		Alive
28	+			+			+				+	+		Loss to follow-up
29					Suppurative tonsillitis	+						+		Alive
30	+	+	+			+					+	+		Alive
31	+	+, RV		+		+	+	+		AIHA	+, Food allergy	+		Died at 2.5 y due to intracranial hemorrhage
32	+	+, Bacilli					+					+		Died at 2m due to severe infection
33		+		+, Respiratory failure			+					+		Alive
34				+		+						+		Loss to follow-up
35						+						+		Alive
36		+		+	Otitis media						+	+	+	Alive
37				+		+					+	+		Alive
38				+		+					Drug allergy	+		Loss to follow-up
39		+	+		CMV infection	+	+				+, Drug allergy	+		Alive
40			+			+					+	+		Alive
41	+	+			Thrush	+	+				+	+		Loss to follow-up
42		+		+, Respiratory failure		+	+	+			+	+	+	Alive

*AIHA, autoimmune hemolytic anemia; GI, gastrointestinal tract; RV, Rotavirus; URI, upper respiratory tract infection*.

#### Bleeding and Hematological Features

Major bleeding events included skin spots or petechiae (*n* = 27, 64.29%), per-rectal bleeding (*n* = 21, 50.00%), epistaxis (*n* = 7, 16.66%) and intracranial bleeding (*n* = 2, 4.76%). Twelve patients (28.57%) had multiple-system bleeding. Moderate and severe anemia usually associated with per-rectal bleeding (12 patients with per-rectal bleeding out of 15 patients with Hb <90 g/L) (Table 2). We found no correlation between platelet count and risk for severe bleeding (*p* = 0.662).

**Table 2:** Mutation type of 42 cases of WAS and other related data.

Patient	Age of onset	Hb (g/L)	Lowest plt (× 10^9^/L)	MPV (fl)	Exon/ intron	Genomic/ cDNA mutation	Predicted codon change	Novel	Maternal carrier status	Score
**NONSENSE**										
1	1m	ND	22	ND	Exon 1	71 C>T	R13X	No	Carrier	3
2	1m	ND	10	5.6	Exon 2	238C>T	Q 80X	No	Carrier	3
3	7m	ND	6	ND	Exon 3	340C>G	Y102X	No	Carrier	3
4	15d	121	39	5.5	Exon 4	436C>T	Q146X	**√**	Carrier	3
5	2m	88	6	9.7	Exon 7	631C>T	R211X	No	ND	3
6	1m	ND	10	ND	Exon 10	961C>T	R321X	No	Carrier	5
7	20d	117	13	ND	Exon 10	1351_1352GC>TT	Q440X	√	Carrier	4
**MISSENSE**										
8	1m	51	18	ND	Exon 1	104T>C	S24P	No	Carrier	5
9	4m	53	22	5.3	Exon 2	251T>C	C73R	No	ND	4
10	5d	ND	12	ND	Exon 2	252G>A	C73Y	No	Carrier	3
11	2m	99	19	4.8	Exon 2	257G>A	V75M	No	ND	2
12	3m	91	15	5.8	Exon 2	279C>A	S82Y	√	ND	3
13	2d	108	26	ND	Exon 2	291T>G	R86L	√	Carrier	2
14	4m	114	15	ND	Exon 3	334G>C	E100D	No	Carrier	3
15	1d	84	47	ND	Exon 7	671A>G	D224G	No	Carrier	3
16	8m	108	20	6.6	Exon 12	1453G>A	D485N	No	ND	3
**FRAMESHIFT**										
17	1d	82	15	ND	Exon 1	141_142delTT	F36X	√	Carrier	4
18	1m	79	10	6.3	Exon 3	330dupC	T111HfsX11	**√**	Carrier	3
19	2m	94	19	5.1	Exon 4	466-467insA	R148fsX168	**√**	Carrier	3
20	1d	92	9	ND	Exon 4	473-474insCA	Q158fsX261	√	ND	3
21	3m	ND	26	ND	Exon 4	486delA	P163QfsX98	√	ND	4
22	7d	80	24	5.2	Exon 7	600delC	P189fsX260	No	Carrier	3
23	1m	78	36	5.8	Exon 7	621-622delGG	G196fsX205	No	Carrier	3
24	8m	88	20	5	Exon 7	681-693ins“CAGCAC CTGGACC”	p220fsX225	No	Carrier	4
25	3d	68	18	5.5	Exon 10	1126delA	R364fsX444	No	ND	4
26	1d	80	22	ND	Exon 10	1305_1306insG	G424fsX494	√	Carrier	3
27	10m	102	6	7.6	Exon 10	1329delG	G432fsX444	√	Carrier	4
**SPLICE SITE**										
28	1m	121	56	ND	Intron 1	IVS1-1G>A	predicted aberrant spicing	**√**	Carrier	3
29	1m	114	11	7.6	Intron 5	IVS5-1G>C	predicted aberrant spicing	**√**	Carrier	3
30	2m	98	15	5.5	Intron 7	IVS7-1G>A	Predicted splice error	No	Carrier	4
31	1d	68	10	7.6	Intron 7	IVS7-1delG	predicted splicing error	No	Carrier	5
32	14d	118	56	ND	Intron 7	IVS7+2T>C	predicted aberrant spicing	**√**	ND	4
33	1m	60	9	6.9	Intron 8	IVS8-1G>A	predicted aberrant spicing	**√**	No	4
34	2d	81	14	6.1	Intron 8	IVS8+1G>A	Predicted aberrant splicing	**√**	Carrier	3
35	1d	90	18	5.6	Intron 8	IVS8+3G>C	predicted aberrant spicing	**√**	Carrier	1
36	1m	104	15	8.2	Intron 8	IVS8+5G>A	predicted aberrant spicing	No	Carrier	3
37	1d	67	5	7.6	Intron 10	IVS10-1G>T	predicted aberrant spicing	√	ND	3
38	2d	139	22	ND	Exon 7	721G>T	G229G, predicted aberrant splicing	√	Carrier	3
**COMPLEX**										
39	5m	125	9	5.1	Exon 1	69G>C+ 96delA	G12A+N21fsX44	No	Carrier	4
40	6m	95	85	ND	Exon 11	1410C>T+ 1455T>A	P459L+M474K	No	Carrier	3
41	2m	94	15	6.4	Large deletion of the whole gene		Predicted no mRNA	/	ND	3
42	5d	ND	2	7.3	Large deletion of the whole gene		Predicted no mRNA	/	ND	4

*ND, not done*.

#### Allergy and Autoimmune Diseases

Twenty-nine patients (69.05%) had eczema, and one patient had a drug allergy. Three patients had autoimmune diseases, among which 2 had autoimmune hemolytic anemia and one autoimmune hemolytic anemia and IgA nephropathy (Table 1).

#### Clinical Scores

Three patients were diagnosed with XLT. A score of 1 was given to 1 patient, and a score of 2 was given to 2 patients. Thirty-nine were diagnosed with classic WAS. A score of 3 was assigned to 24 patients, and a score of 4 was given to 12 patients. Three patients progressed to a score of 5 after developing autoimmune diseases (P6 and P31 had autoimmune hemolytic anemia, P8 had autoimmune hemolytic anemia, and IgA nephropathy) (Table 2).

### Laboratory Examination of WAS/XLT Patients

All patients had low platelet counts vary from 2 to 85 × 10^9^/L. Twenty-five patients had MPV detection among which 16 patients (64.00%) had small platelet size (normal range [6.5–11] fl). There was no correlation between the severity of anemia and the level of platelets (*p* > 0.05).

Eosinophils (EOS) were available in 42 patients, which varied from 140 to 3,210 × 10^6^/L. The median EOS count was 418 × 10^6^/L, and 29 patients (69.05%) had elevated EOS counts (normal range <300 × 10^6^/L).

Data on IgG/A/M and IgE were available in 33 and 20 patients, respectively. Because many patients were misdiagnosed with ITP before WAS genetic testing was done, many patients used IVIG before IgG/A/M detection. Therefore, IgG was analyzed in 14 patients after we discarded this part of patients. Low serum IgG (<4 g/L) was present in 28.57% of patients, while only one (6.67%) had raised IgG (>10 g/L), and 9 patients (64.29%) had normal serum IgG level. IgA was analyzed in 33 patients, 19 of whom (57.58%) had normal IgA. Twelve patients (36.36%) had raised IgA (>0.76 g/L), while low serum IgA (<0.1 g/L) was present in 6.06% of patients. Low serum IgM (<0.5 g/L) was present in 39.39% of patients, while six (18.18%) had raised IgM (>1.25 g/L), and 14 patients (42.42%) had normal serum IgM. Serum IgE was elevated in 70.00% (14/20) of them.

Data on lymphocyte subset were available in 34 patients. Diminished CD3^+^ T lymphocyte count was found in 21 patients (61.76%), and reversed CD4/CD8 ratio was found in 16 patients (47.05%). Sixteen patients (47.05%) had raised CD19^+^B cell count.

### Association Between Lymphocyte Subsets and Was Clinical Scores

We divided the 34 WAS patients into four groups according to clinical score and then examined the relationships between the percentiles and of lymphocyte subsets and clinical scores. Though there was no correlation between the lymphocyte subset and WAS clinical score, patients with a clinical score of 1–2 had significantly higher percentages of CD3^+^ T cells than those with a score of 5 (*p* < 0.05).

### Mutations of WAS/XLT Patients

We identified and characterized at total of 42 unique WASP mutations, including 2 large deletions of the whole gene in 42 patients with WAS/XLT from 42 unrelated families of which 19 had not been previously reported. Genetic investigations of the maternal carrier status were carried out in 30 mothers and 29 were confirmed to be heterozygous for the mutation detected in their children.

**Figure 1:**
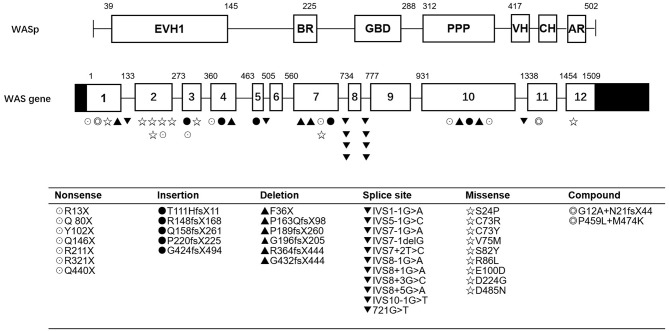
WASP mutations identified in 42 patients. EVH1, Ens/VASP homology 1 domain; BR, basic region; GBD, GTPase bending domain; PPPP, proline-rich region; VH, verprolin homology; CH, cofilin homology; AR, acidic region.

As shown in Table 2 and Figure 1, the most common mutations were splice site mutations (*n* = 11), situated preferentially in introns 1, 5, 7, 8, and 10, followed by missense mutations (*n* = 9), distributed predominantly in exon 2, which encodes for the EVH1 domain. Nonsense mutations (*n* = 7) were located throughout the WAS gene. Deletion (*n* = 6) and insertion mutations (*n* = 5) resulting in frame shifts and early termination of transcription were also distributed throughout the WAS gene. Complex mutation made up 9.52% of the mutations identified, and 2 patients had large deletions of the whole gene, predicted to result in no protein expression.

**Figure 2:**
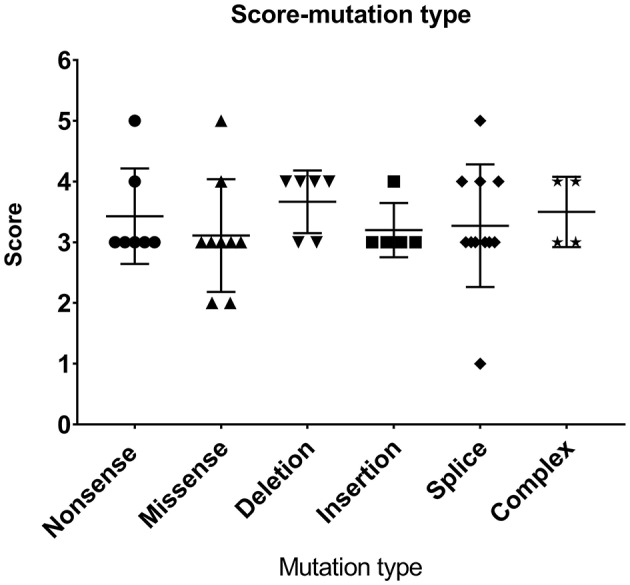
Relationship between mutation type and clinical scores.

Most patients with XLT (2/3, P11, and P13) had missense mutations, which were mostly located in exon 2. Patients with classic *WAS* had non-sense mutations, deletions/insertions mutations, or splice site mutations. Exon 2 was also the hotspot of missense mutations (5/9, 55.56%). Intron 7 and Intron 8 were the hotspots of splice site mutations (8/11, 72.72%) (Figure 1). We found no correlation between mutation type and clinical score (*p* > 0.05, Figure 2).

### Treatment and Outcomes of WAS/XLT Patients

Thirty-one patients (73.81%) were treated with IVIG because many patients (28, 66.67%) were misdiagnosed with ITP, confirmed by bone marrow smear, before WAS gene mutation was found. Six patients (19.35%) initially respond to IVIG, but platelets dropped again a few days later. No patient underwent splenectomy.

As shown in Table 3, HSCT was performed in eight patients. Age at transplantation ranged from 0.5 to 4 year (median, 1 year). The hematopoietic stem cell source was peripheral-blood stem cells (PBSCs) in 6 patients, cord blood in P21 and cord blood plus PBSCs in P36 for a mismatched related donor (5/10). P22 underwent HLA-matched sibling PBSC transplantation, the donor being his dizygotic twin brother. Four patients (P5, P11, P13, and P22) were fully matched HLA from unrelated donor. Two (P6 and P42) were mismatched HLA at one locus (9/10), and the other two patients (P21 and P36) were mismatched HLA at three or even five loci. A conditioning regimen containing busulfan (20 mg/kg total dose), cyclophosphamide (200 mg/kg total dose), and antithymocyte globulin (ATG) (15 mg/kg total dose) was used in the patients. All patients received cyclosporine and methotrexate for GVHD prophylaxis. Acute grade I-II GVHD occurred in six patients (P5, P6, P11, P13, P21), while P36 and P42 had grade III GVHD. All these patients exhibited full chimerisms after transplantation and have survived thus far.

**Table 3:** Clinical data of 8 patients with HSCT.

**Patient**	**Age at HSCT**	**Type of HSCT**	**HLA match**	**Conditioning regimen**	**Prevention of GVHD**	**GVHD (grade)**	**Chimerism (Donor cells)**	**Time of Neutrophil engraftment**	**Time of PLT engraftment**	**Current age (years post-HSCT)**	**Outcome and status at last follow-up**
5	4 y	MUD, PBSC	10/10	Bu, CTX, ATG	Cyc+IVIG	Liver(II)	100%	D+14	D+36	9 y (5 y)	Alive and well
6	3.5 y	Partially MUD, PBSC	9/10	Bu, CTX, ATG	Cyc+IVIG	Skin(II)	99.93%	D+11	D+13	5 y (1.5 y)	Alive and well
11^*^	5 y	MUD, PBSC	10/10	Bu, CTX, ATG	Cyc+IVIG	Skin(I)	97.4%	D+11	D+13	9 y (4yeras)	Alive and well
13^*^	1 y	MUD, PBSC	10/10	Bu, CTX, ATG	Cyc+IVIG	Skin(I)	99.52%	D+12	D+16	9 y (8 y)	Alive and well
21	1 y	Partially MUD, Cord blood	7/10	Bu, CTX, ATG	Cyc+IVIG	Skin(II)	100%	D+14	D+22	2 y (1 y)	Alive and well
22	6 m	MSD (twin), PBSC	10/10	Bu, CTXM, ATG	Cyc+IVIG	Nil	100%	D+11	D+13	12 y (11.5 y)	Alive and well
36	9 m	MMRD(father) +cord blood	5/10	Bu, CTX, ATG	Cyc+IVIG	Skin(III) Intestine(III)	98.16%	D+11	D+28	3 y (2 y)	Alive and well
42	11 m	Partially MUD, PBSC	9/10	Bu, CTX, ATG	Cyc+IVIG	Skin(III)	99.43%	D+11	D+16	2 y (1 y)	Alive and well

ATG, anti-thymocyte globulin; Bu, busulphan; Cyc, Cyclosporin; CTX, Cyclophosphamide; GVHD, graft-versus-host disease; HSCT, hematopoietic stem cell transplantation; IVIG, Intravenous immunoglobulin; MRD, matched related donor; MMRD, mismatched related donor; MSD, matched sibling donor; MUD, matched unrelated donor; PBSC, peripheral blood stem cells

* *XLT*.

*Neutrophil engraftment was defined as >500 granulocytes per microliter and platelet engraftment as stable or increasing platelet count >50 × 10^9^/L without further necessity of substitution*.

Five patients out of 42 had died at follow-up. Three died of severe infection (P7, P17, and P32), and two died of intracranial hemorrhage (P18 and P31). Eight patients were lost to follow-up for various reasons, and the other 29 patients were still alive but 21 patients still suffering from recurrent infections and bleeding.

### Comparison Between WAS/XLT Patients and ITP Patients

**Figure 3:**
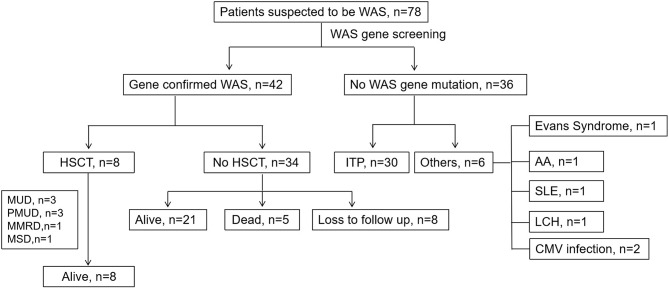
The clinical course of patients suspected to have WAS. MUD, matched unrelated donor; PMUD, partially matched unrelated donor; MSD, matched-sib donor; MMRD, mismatched related donor; ITP, idiopathic thrombocytopenic purpura; SLE, systemic lupus erythematosus; LCH, Langerhans cell histiocytosis; HSCT, hematopoietic stem cell transplantation.

The rest of 36 patients suspected to have WAS finally did not have a WAS gene mutation. Thirty (83.33%) of them were diagnosed with ITP. Of the other 6 patients, two had symptoms secondary to CMV infection. The other four were diagnosed with Evans syndrome, Langerhans cell histiocytosis (LCH), aplastic anemia and systemic lupus erythematosus (SLE), respectively (Figure 3). We compared the clinical data and related laboratory results between the WAS/XLT and ITP patients.

Eczema is one of the characteristic findings that originally differentiated WAS/XLT from ITP. Twenty-eight patients in the WAS/XLT group had eczema (28/42, 66.67%), while only 8 patients in ITP group had eczema (8/30, 26.67%) (*p* = 0.001).

**Figure 4:**
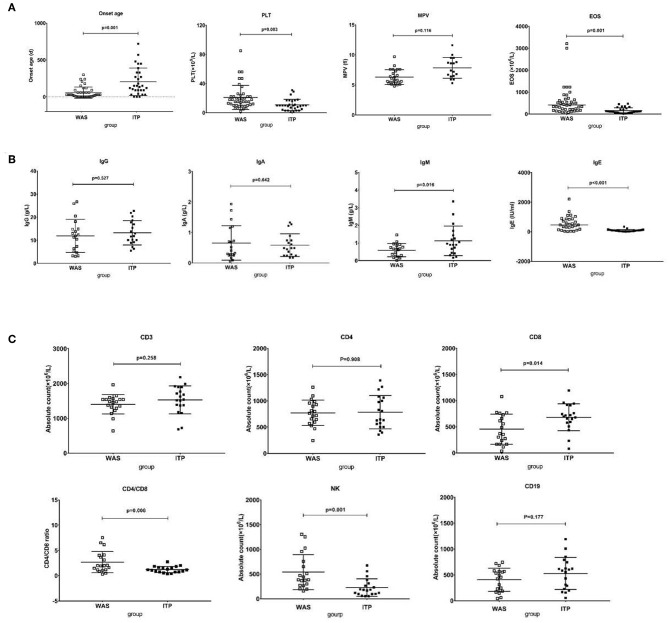
Comparison between WAS patients and ITP patients. **(A)** The comparison of onset age, PLT, EOS, and MPV between the two groups. The differences in the former three were significant (*p* < 0.01). **(B)** The comparison of IgG, IgA, IgM, and IgE between the two groups. The differences in the latter two were significant (*p* < 0.05). **(C)** The comparison of lymphocytes subsets between the two groups. The differences in CD8+ T cell count, the ratio of CD4/CD8 and NK cell count were significant (*p* < 0.05).

As shown in Figure 4, the median onset age of WAS/XLT (*n* = 42) was far earlier than that of ITP (*n* = 30) [1 month (1 day−10 months) vs. 4.8 months (1 day−2 years), *p* = 0.001]. However, there was no difference in diagnosis age between the two groups (*p* = 0.115). The PLT level of WAS/XLT was higher than that of ITP (20.98 ± 2.55 vs. 10.83 ± 1.39, *p* = 0.003). Data on MPV in WAS/XLT and ITP were available in 25 and 17 patients, respectively, because the MPV could not be detected when the PLT count was very low. Though the MPV in WAS/XLT patients was smaller than that in ITP patients (6.31 ± 1.15 vs. 7.85 ± 0.92), the difference was not statistically significant (*p* = 0.116). The median EOS count in WAS/XLT patients was higher than that in ITP patients (410 vs. 110, *p* = 0.001).

Considering the differences in immunoglobulin levels and lymphocyte subset distributions in different age groups, we examined the differences in the immunoglobulin level and absolute counts of distinct lymphocyte subsets between 19 WAS patients and 19 age-matched ITP patients. Paired *t*-test was used to compare the data between the two groups.

The IgE level in the WAS/XLT group was much higher than that in the ITP group (602.6 1 ± 121.91 vs. 81.45 ± 19.02, *p* < 0.001). The IgM level in the WAS group was lower than that in the ITP group (0.59 ± 0.08 vs. 1.12 ± 0.19, *p* = 0.016). The differences in IgG and IgA between the two groups were not statistically significant (Figure 4).

There was no significant difference in total lymphocyte count between the two groups (2450 ± 85.83 vs. 2285 ± 86.95, *p* = 0.602). The absolute number of CD8^+^ T cells was significantly lower in the WAS patients than that in ITP patients (453.42 ± 65.97 vs. 682.41 ± 59.41, *p* = 0.014). The ratio of CD4/CD8 was higher in the WAS patients than that in the ITP patients (2.69 ± 0.48 vs. 1.22 ± 0.14, *p* = 0.006). The absolute number of NK cells in the WAS group were higher than that in the ITP group (540.62 ± 81.41 vs. 226.53 ± 40.65, *p* = 0.001). The differences between the two groups in CD3^+^, CD4^+^ T cells, and CD19^+^ B cells were not statistically significant (Figure 4).

The above analysis is based on univariate analysis. In order to verify the difference between the two groups again, we used multivariate analysis by Sidak's multiple comparisons test and found that the differences between the two groups in onset age, PLT level, EOS counts, IgE level, CD8^+^ T cell count and NK cell count were also statistically significant (*p* = 0.005, 0.023, 0.001, 0.001, 0.045, 0.008, respectively).

## Discussion

In China, the diagnosis of WAS has improved substantially over the last decade, and many cases have been reported already (23, 24), although a much higher number of cases had been expected. Typical WAS can be straightforwardly diagnosed in children with positive family histories and typical signs. However, the diagnosis may be delayed in children without family histories or with milder phenotypes, such as XLT, particularly among infants. Furthermore, WAS/XLT is ultimately diagnosed in 7% of patients with conditions that are often mistaken for ITP (25). Many WAS/XLT patients are initially diagnosed and treated for ITP, which could delay the life-saving definitive therapy, as the earlier the HSCT treatment is performed, the higher the survival rate is.

As far as we know, there are very limited reports on the comparison of clinical and laboratory characteristics between WAS and ITP. The differentiation of WAS and ITP has only been studied by Sokolic et al. (26). They observed that WAS patients have a low immature platelet fraction (IPF) compared with ITP and concluded that IPF is a convenient and readily available platelet parameter to differentiate these two syndromes. This is very valuable and useful for pediatricians. However, this detection method is not widely used in most developing countries which limited its application. By comparing the clinical and laboratory data of WAS/XLT and ITP patients, we also found some clues to distinguish them before genetic diagnosis. And these findings may be complementary to that of Candotti and colleagues.

### Onset Age

WAS is a congenital hereditary disease, so the onset age may be in early childhood. The median onset age of our 42 patients was 1 month (range: 1 day−10 months) which is similar to that in Zhao's study. The median onset age of their patients was 0.5 month (range:1 day−51.6 months) (*n* = 81) (24). The onset age of XLT seemed to be later than that of classic WAS (2.0–74.6 years, *n* = 173) (27). In addition, the onset age of WAS patients assigned a score of 5 seemed to be earlier (median: 6.2 months, range: 2–22.5 months, *n* = 26) (28). Our study found that the onset age was not correlated with the score. Due to the limited number of XLT patients in our cohort, we did not compare that between XLT and classic WAS patients. However, we compared the onset age of WAS/XLT patients with that of ITP patients. The onset age of WAS/XLT was far earlier than that of ITP in our study. In childhood, the peak onset age for ITP is 2–4 years (29). Though there are still infants, even newborns, with ITP, patients with early-onset ITP, especially <1-year-old, should undergo WAS genetic screening as early as possible.

### MPV and PLT Level

MPV used to be a useful tool to differentiate WAS/XTL and ITP. However, microthrombocytopenia may occasionally not be present, and some WAS patients have been found to have normal MPV (15, 16) or macrothrombocytopenia (2, 17). Though the MPV in WAS/XLT patients in our study was smaller than that in ITP patients, the difference was not statistically significant. So, MPV may only be of some significance in prompting the diagnosis of WAS/XLT, but it cannot be used as a reliable diagnostic clue. Interestingly, we found that the PLT level of WAS/XLT patients was higher than that of ITP. From the point of view of pathogenesis, both syndromes have impaired platelet production and increased platelet destruction. The mechanisms needed to be clarified.

No clear correlation between platelet count and risk for severe bleeding in WAS/XLT patients was found in our study. This was also consistent with a retrospective analysis in a large cohort of XLT patients (27).

### Eczema EOS and IgE

Eczema is one of the typical findings that differentiate WAS from ITP. However, XLT patients have either mild and transient eczema or none at all (30). Our study found that the incidence of eczema in WAS/XLT patients was significantly higher than that in ITP patients. Eczema can still be used as an effective clinical clue to distinguish them. It has been hypothesized that defective chemotaxis of DCs and Langerhans cells plays a role in the local generation of antigen-specific T cells that are responsible for the development of eczema (31).

The EOS count and IgE level in the WAS/XLT group were much higher than those in the ITP group. Although higher IgE may represent a possible cause of eczema, the correlation between increased IgE and eczema has not yet been demonstrated. WAS T-cell lines have a selective defect in TH1 cytokine production, and WASP-deficient mice develop a skewed TH2 phenotype, supporting the hypothesis that eczema and high IgE in patients with WAS are related to a TH2 imbalance (32, 33). However, the mechanism driving the eosinophilia is unclear (34). Elevated level of EOS counts and IgE can be a clinical clue to distinguish WAS/XLT and ITP. Further study is needed to clarify the mechanisms of these perturbations.

### Lymphocyte Subsets

A distribution disorder of immune subsets was found in our study. We found increased NK cell numbers but diminished CD8^+^T lymphocytes in WAS/XLT patients.

The data are highly informative in revealing a significant decrease in T lymphocytes, especially CD8^+^ T lymphocytes. This is consistent with the early onset of recurrent infections in patients with WAS. As we known, CD8^+^ T cells play critical roles in in anti-virus efforts and cancer surveillance. A significant proportion of patients with WAS present chronic viral infections, most commonly involving members of the herpes virus family. This is associated with an intrinsic defect in the ability of CD8^+^ T cells to kill target cells presenting viral epitopes combined with a reduced priming due to the inability of DCs to produce type I IFN. Additionally, WAS patient CTLs display a reduced cytotoxicity against tumoral B cell lines that can be rescued by restoring WASP expression by means of a lentiviral vector (35). One study suggested that the patients' decreased cell numbers were due to deficient output, possibly resulting from impaired lymphocyte maturation (36). The findings suggest “enhancement of lymphocyte output” as a potential clinical target for future therapeutic interventions.

### Mutations in WAS/XLT

We identified 42 mutations, including 2 large deletions of the whole gene, of which 19 had not been previously reported. The mutation types were similar to those reported by others (24, 37, 38). Ochs (37) reported 141 unique mutations from 227 WAS/XLT families, mostly Europeans and Americans, with a total of 262 affected members, and identified 5 mutational hotspots in the WASP gene: 168C>T, 290C>N/291G>N, IVS+5g>a, 665C>T, and IVS8+1g>n. The J Project Study Group in European reported 87 affected males and 48 carrier females from 77 WAS families and identified 62 unique mutations, including 17 novel sequence variants. They identified that the most commonly affected amino acids were Arg86 and Asp224 (38). Whereas, Liu et al. (24) in China identified 60 unique mutations from 75 unrelated families with a total of 81 affected members and found eight hotspots including four point mutations (155C>T,168C>N, 290C>N/291G>N, and 665C>T), one frameshift mutation within the coding region (797delC), and three splice site mutations (IVS1+1g>n, IVS3+1g>n, IVS7+2t>c). We can see that 168C>T, 290C>N/291G>N, and 665C>T were the common hotspots regardless ethnic origins. No mutational hotspots were found in our study. The reason for this may be the relatively small number of patients.

Most patients with XLT (2/3, P11, and P13) in our study had missense mutations, which were mostly located in exon 2. This is consistent with other reports. Missense mutations in exons 1 and 2 of the gene (affecting the EVH1 domain) are most often identified in patients with a milder variant of the disease, XLT (39).

Two large deletions of the whole gene (P41 and P42) were identified in our study. To the best of our knowledge, only one case of whole-exon deletion has been reported in China, and two cases in the world (40). The onset age of P41 was 2 months old, and his disease manifested as intestinal infection, recurrent thrush, eczema, and skin hemorrhage, and hemangioma and he was given a score of 3. He had positive family history. The lowest PLT was 15 × 10^9^/L and the MPV was 6.4fl. EOS counts varied from 1.78 to 3.0 × 10^9^/L, and IgE was 172 IU/ml. Serum IgA and IgG were elevated, which may be associated with intravenous Ig therapy. IgM increased significantly, and this may have been associated with intestinal infection and thrush. The age of onset of P42 was 5 days after birth, with severe bleeding (skin, digestive tract, and nasal cavity) and infections. He even had respiratory failure due to severe pneumonia that required ventilator-assisted ventilation. The lowest PLT was 2 × 10^9^/L and the MPV was 7.3 fl. Both patients had reduced T cell counts whereas B lymphocyte numbers increased, which is consistent with other studies (37, 40). Neither autoimmune or tumor diseases were identified in either patients, which may be associated with the young age of the patients, and unfortunately, P41 was lost to follow-up. P42 had HSCT and still alive and be well-being. It seemed that patients with large deletion of the whole gene would had severe symptoms, but it still need further study due to the limited cases identified and the short duration of the disease. Furthermore, the mechanism needs to be clarified in further study.

### Does Patients With XLT Require HSCT?

Whether XLT patients require HSCT is a difficult problem to decide. Three patients with XLT (P11 and P13) in our cohort underwent HSCT and survived well. P11 had a missense mutation (257G>A, V75M), which had been reported by Kolluri et al. (41). Their patient was diagnosed with WAS at the age of 16 and underwent splenectomy for hypersplenism due to long-term thrombocytopenia at the age of 10. P13 had family members who died of severe infections. For these reasons, both of them were advised to proceed HSCT. Previous studies have shown that only 35% of WASp-negative patients, especially those aged below 2 years, were found with clinical scores ranging from 1.0 to 2.5, indicative of milder phenotypes; noticeably, an even lower proportion (7%) of WASp-negative patients had mild phenotypes when evaluated at older ages (>2 year) (37).This provides evidence that a mild XLT phenotype may gradually evolve to a more severe disease, and this evolution may take several years. If economic conditions permit, HSCT might be considered a viable option for patients with XLT if a human leukocyte antigen-identical donor can be identified. But it still has to be decided on an individual patient basis.

In the present study, no tumor diseases were identified, which may be associated with the young age of the patient and the short duration of the disease. Limitations of this study include its small sample size and relatively short follow-up time.

**Figure 5:**
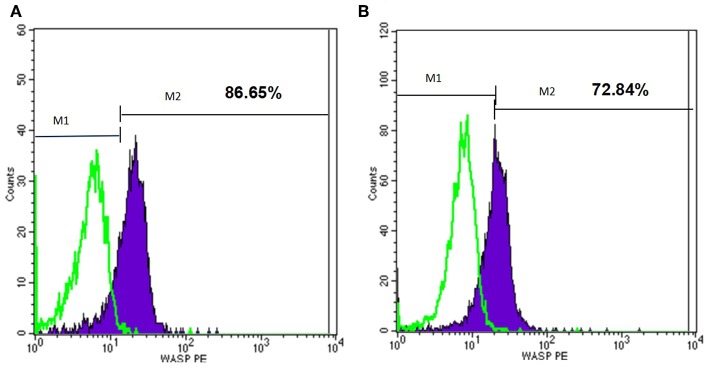
Flow cytometry analysis of the WASp expression of one patient. M1, isotype-matched control mouse IgG2a mAb stained; M2, purified mouse anti-human WASp mAb stained. **(A)** WASp expression level of normal control. **(B)** WASp expression level of one patient (P22) with *WAS* gene mutation.

At the beginning, we used flow cytometry to detect WASp in several patients, but it did not match the results of gene detection. For example, P22 had mutation of 600delC in Exon 7, however, his WASP expression was almost normal (Figure 5). The reason for this may be that some districts in WAS gene seem to be more important for the WASp expression and function. It is reported that patients with missense mutations allowing expression of mutated WASp and those with splice anomalies, which result in generation of multiple products, including normal WASp (24). Therefore, the latter patients did not undergo this test. And this may miss some patients who had reduced or absent expression of WASP but without the mutation in exon (and adjuscent intron) region. This is also one of the shortcomings of this study.

The primay purpose of this study is to find clues to distinguish WAS from ITP clinically before genetic diagnosis because we found many gene confirmed WAS patients had been initially diagnosed as ITP and been treated as ITP which delayed the effective treatment of WAS. But we are deeply aware that if these differences that can be used to distinguish patients with only bleeding and/or thrombocytopenia or XLT and WAS, it will be of greater significance to clinicians. But due to the limited cases of XLT in our cohort (only 3 patients diagnosed to have XLT and also only these 3 patients had thrombocytopenia and/or bleeding as the only clinical presentation), It is difficult to make statistical analysis. We need more XLT patients to further confirm this in our future study.

## Conclusion

The *WAS* gene diagnosis should be considered in all males with ITP-like features, especially for patients with a very early onset age, decreased MPV (<6.5 fl), higher EOS counts and elevated IgE level, increased NK cell number, diminished CD8^+^T lymphocyte count.

## Ethics Statement

This study was carried out in accordance with the recommendations of Children's National Medical Center, Shanghai Children's Medical Center, Shanghai Jiao Tong University School of Medicine with written informed consent from all subjects. All subjects gave written informed consent in accordance with the Declaration of Helsinki. The protocol was approved by the Children's National Medical Center, Shanghai Children's Medical Center, Shanghai Jiao Tong University School of Medicine.

## Author Contributions

YJ was responsible for data collection and article writing. JW was responsible for article revising. TC and JC were responsible for project design, patients recruitment, article conception, and revising.

### Conflict of Interest Statement

The authors declare that the research was conducted in the absence of any commercial or financial relationships that could be construed as a potential conflict of interest.

## References

[1] Massaad MJ Ramesh N Geha RS. Wiskott-Aldrich syndrome: a comprehensive review. Ann N Y Acad Sci. (2013). 1285:26–43. 10.1111/nyas.12049 23527602

[2] Skoric D Dimitrijevic A Cuturilo G Ivanovski P. Wiskott-Aldrich syndrome with macrothrombocytopenia. Indian Pediatr. (2014) 51:1015–6. 10.1007/s13312-014-0550-5 25560165

[3] Wiskott A Familiarer, angeborener Morbus Werlhofii? Monatsschr. Kinderheilkd. (1937) 68:212–6.

[4] Aldrich RA Steinberg AG Campbell DC. Pedigree demonstrating a sex-linked recessive condition characterized by draining ears, eczematoid dermatitis and bloody diarrhea. Pediatrics. (1954) 13:133–9. 13133561

[5] Derry JM Ochs HD Francke U. Isolation of a novel gene mutated in Wiskott-Aldrich syndrome. Cell. (1994) 78:635–44. 10.1016/0092-8674(94)90528-2 8069912

[6] Notarangelo LD Miao CH Ochs HD Wiskott-aldrich syndrome. Curr Opini Hematol. (2008) 15:30–6. 10.1097/MOH.0b013e3282f30448 18043243

[7] Snapper SB Rosen FS Mizoguchi E Cohen P Khan W Liu CH Wiskott-Aldrich syndrome protein-deficient mice reveal a role for WASP in T but not B cell activation. Immunity. (1998) 9:81–91. 10.1016/S1074-7613(00)80590-7 9697838

[8] Villa A Notarangelo L Macchi P Mantuano E Cavagni G Brugnoni D. X-linked thrombocytopenia and Wiskott-Aldrich syndrome are allelic diseases with mutations in the WASP gene. Nat Genet. (1995) 9:414–7. 10.1038/ng0495-414 7795648

[9] Devriendt K Kim AS Mathijs G Frints SG Schwartz M Van Den Oord JJ. Constitutively activating mutation in WASP causes X-linked severe congenital neutropenia. Nat Genet. (2001) 27:313–7. 10.1038/85886 11242115

[10] Griffith LM Cowan MJ Notarangelo LD Kohn DB Puck JM Shearer WT. Primary immune deficiency treatment consortium (PIDTC) update. J Allergy Clin Immunol. (2016) 138:375–85. 10.1016/j.jaci.2016.01.051 27262745 PMC4986691

[11] Strom TS. The thrombocytopenia of WAS: a familial form of ITP? Immunol Res. (2009) 44:42–53. 10.1007/s12026-008-8069-2 18854955

[12] Karalexi MA Tzanoudaki M Fryganas A Gkergki A Spyropoulou D Papadopoulou A. Wiskott-aldrich syndrome misdiagnosed as immune thrombocytopenic purpura: a case report. J Pediatr Hematol Oncol. (2018) 40:240–2. 10.1097/MPH.0000000000000949 28859046

[13] Kaneko R Yamamoto S Okamoto N Akiyama K Matsuno R Toyama D Wiskott-Aldrich syndrome that was initially diagnosed as immune thrombocytopenic purpura secondary to a cytomegalovirus infection. SAGE. Open Med Case Rep. (2018) 6:2050313X17753788 10.1177/2050313X17753788 PMC5768273 29348920

[14] Worth AJ Thrasher AJ. Current and emerging treatment options for Wiskott-Aldrich syndrome. Exp Rev Clin Immunol. (2015) 11:1015–32. 10.1586/1744666X.2015.1062366 26159751

[15] Yoonessi L Randhawa I Nussbaum E Saharti S Do P Chin T. Wiskott-aldrich syndrome: description of a new gene mutation with normal platelet volume. J Pediatr Hematol Oncol. (2015) 37:515–8. 10.1097/MPH.0000000000000392 26241726

[16] Baharin MF Dhaliwal JS Sarachandran SV Idris SZ Yeoh SL. A rare case of Wiskott-Aldrich Syndrome with normal platelet size: a case report. J Med Case Rep. (2016) 10:188. 10.1186/s13256-016-0944-1 27356510 PMC4928304

[17] Bastida JM Del Rey M Revilla N Benito R Perez-Andrés M González B. Wiskott-Aldrich syndrome in a child presenting with macrothrombocytopenia. Platelets. (2017) 28:417–20. 10.1080/09537104.2016.1246715 27885891

[18] Ochs HD Filipovich AH Veys P Cowan MJ Kapoor N. Wiskott-Aldrich syndrome: diagnosis, clinical and laboratory manifestations, and treatment. Biol Blood Marrow Transplant. (2009) 15(Suppl 1):84–90. 10.1016/j.bbmt.2008.10.007 19147084

[19] Zhu Q Watanabe C Liu T Hollenbaugh D Blaese RM Kanner SB. Wiskott-Aldrich syndrome/X-linked thrombocytopenia: WASP gene mutations, protein expression, and phenotype. Blood. (1997) 90:2680–9. 9326235

[20] Imai K Nonoyama S Ochs HD. WASP (Wiskott-Aldrich syndrome protein) gene mutations and phenotype. Curr Opin Allergy Clin Immunol. (2003) 3:427–36. 10.1097/00130832-200312000-00003 14612666

[21] Yamada M Ohtsu M Kobayashi I Kawamura N Kobayashi K Ariga T. Flow cytometric analysis of Wiskott-Aldrich syndrome (WAS) protein in lymphocytes from WAS patients and their familial carriers. Blood. (1999) 93:756–57. 10215346

[22] Chan KW Lee TL Chung BH Yang X Lau YL. Identification of five novel WASP mutations in Chinese families with Wiskott-Aldrich syndrome. Hum Mutat. (2002) 20:151–2. 10.1002/humu.9048 12124997

[23] Lee PP Chen TX Jiang LP Chen J Chan KW Lee TL. Clinical and molecular characteristics of 35 Chinese children with Wiskott-Aldrich syndrome. J Clin Immunol. (2009) 29:490–500. 10.1007/s10875-009-9285-9 19308710

[24] Liu DW Zhang ZY Zhao Q Jiang LP Liu W Tu WW. Wiskott-Aldrich syndrome/X-linked thrombocytopenia in China: clinical characteristic and genotype-phenotype correlation. Pediatr Blood Cancer. (2015) 62:1601–8. 10.1002/pbc.25559 25931402

[25] Bryant N Watts R. Thrombocytopenic syndromes masquerading as childhood immune thrombocytopenic purpura. Clin. Pediatr. (2011) 50:225–30. 10.1177/0009922810385676 21098529

[26] Sokolic R Oden N Candotti F. Assessment of immature platelet fraction in the diagnosis of wiskott-aldrich syndrome. Front Pediatr. (2015) 3:49. 10.3389/fped.2015.00049 26082919 PMC4450723

[27] Albert MH Bittner TC Nonoyama S Notarangelo LD Burns S Imai K. X-linked thrombocytopenia (XLT) due to WAS mutations: clinical characteristics, long-term outcome, and treatment options. Blood. (2010) 115:3231–8. 10.1182/blood-2009-09-239087 20173115

[28] Mahlaoui N Pellier I Mignot C Jais JP Bilhou-Nabéra C Moshous D. Characteristics and outcome of early-onset, severe forms of Wiskott-Aldrich syndrome. Blood. (2013) 121:1510–6. 10.1182/blood-2012-08-448118 23264593

[29] George JN Woolf SH Raskob GE. Idiopathic thrombocytopenic purpura: a guideline for diagnosis and management of children and adults. Am Soc Hematol. (1998) 30:38–44. 10.3109/07853899808999383 9556088

[30] Imai K Morio T Zhu Y Jin Y Itoh S Kajiwara M. Clinical course of patients with WASP gene mutations. Blood. (2004) 103:456–64. 10.1182/blood-2003-05-1480 12969986

[31] Thrasher AJ Jones GE Kinnon C Brickell PM Katz DR. (1998). Is Wiskott–Aldrich syndrome a cell trafficking disorder? Immunol Today. 19:537–9. 10.1016/S0167-5699(98)01350-4 9864941

[32] Grimbacher B Belohradsky BH Holland SM. Immunoglobulin E in primary immunodeficiency diseases. Allergy. (2002) 57:995–1007. 10.1034/j.1398-9995.2002.02168.x 12358995

[33] Trifari S Sitia G Aiuti A Scaramuzza S Marangoni F Guidotti LG. Defective Th1 cytokine gene transcription in CD4+ and CD8+ T cells from Wiskott-Aldrich syndrome patients. J Immunol. (2006) 177:7451–61. 10.4049/jimmunol.177.10.7451 17082665

[34] Williams KW Milner JD Freeman AF Eosinophilia associated with disorders of immune deficiency or immune dysregulation. Immunol Allergy Clin North Am. (2015) 35:523–44. 10.1016/j.iac.2015.05.004 26209898 PMC4688016

[35] Cotta-de-Almeida V Dupré L Guipouy D Vasconcelos Z. Signal Integration during T lymphocyte activation and function: lessons from the wiskott-aldrich syndrome. Front Immunol. (2015) 6:47. 10.3389/fimmu.2015.00047 25709608 PMC4321635

[36] Park JY Kob M Prodeus AP Rosen FS Shcherbina A Remold-O'Donnell E. Early deficit of lymphocytes in Wiskott-Aldrich syndrome: possible role of WASP in human lymphocyte maturation. Clin Exp Immunol. (2004) 136:104–10. 10.1111/j.1365-2249.2004.02409.x 15030520 PMC1809006

[37] Jin Y Mazza C Christie JR Giliani S Fiorini M Mella P. Mutations of the Wiskott-Aldrich Syndrome Protein (WASP): hotspots, effect on transcription, and translation and phenotype/genotype correlation. Blood. (2004) 104:4010–9. 10.1182/blood-2003-05-1592 15284122

[38] Gulácsy V Freiberger T Shcherbina A Pac M Chernyshova L Avcin T. Genetic characteristics of eighty-seven patients with the Wiskott-Aldrich syndrome. Mol Immunol. (2011) 48:788–92. 10.1016/j.molimm.2010.11.013 21185603

[39] Notarangelo LD Mazza C Giliani S D'Aria C Gandellini F Ravelli C. Missense mutations of the WASP gene cause intermittent X-linked thrombocytopenia. Blood. (2002) 99:2268–9. 10.1182/blood.V99.6.2268 11877312

[40] He X Zou R Zhang B You Y Yang Y Tian X Whole Wiskott-Aldrich syndrome protein gene deletion identified by high throughput sequencing. Mol Med Rep. (2017) 16:6526–31. 10.3892/mmr.2017.7416 28901403 PMC5865821

[41] Kolluri R Shehabeldin A Peacocke M Lamhonwah AM Teichert-Kuliszewska K Weissman SM. Identification of WASP mutations in patients with Wiskott-Aldrich syndrome and isolated thrombocytopenia reveals allelic heterogeneity at the WAS locus. Hum Mol Genet. (1995) 4:1119–26. 10.1093/hmg/4.7.1119 8528198

